# The Effects of La Doping on the Crystal Structure and Magnetic Properties of Ni_2_In-Type MnCoGe_1−x_La_x_ (x = 0, 0.01, 0.03) Alloys

**DOI:** 10.3390/ma14143998

**Published:** 2021-07-16

**Authors:** Yifei Bi, Wei He, Tonghan Yang, Weining Wu, Jingxian Wen, Xi Yu, Feikuo Chen

**Affiliations:** School of Resources, Environment and Materials, Guangxi University, Nanning 530004, China; byf960523@163.com (Y.B.); yangthan199@163.com (T.Y.); 18582018748@163.com (W.W.); wjxfh@126.com (J.W.); gxuyuxi@163.com (X.Y.); chenfeikuo@163.com (F.C.)

**Keywords:** MnCoGe alloy, rare earth doping, Rietveld full spectrum fitting, crystal structure, magnetic properties

## Abstract

In this experiment, a series of MnCoGe_1−x_La_x_ (x = 0, 0.01, 0.03) alloy samples were prepared using a vacuum arc melting method. The crystal structure and magnetic properties of alloys were investigated using X-ray diffraction (XRD), Rietveld method, physical property measurement system (PPMS), and vibrating sample magnetometer (VSM) analyses. The results show that all samples were of high-temperature Ni_2_In-type phases, belonging to space group P6_3_/mmc (194) after 1373 K annealing. The results of Rietveld refinement revealed that the lattice constant and the volume of MnCoGe_1−x_La_x_ increased along with the values of La constants. The magnetic measurement results show that the Curie temperatures (T_C_) of the MnCoGe_1−x_La_x_ series alloys were 294, 281, and 278 K, respectively. The maximum magnetic entropy changes at 1.5T were 1.64, 1.53, and 1.56 J·kg^−1^·K^−1^, respectively. The respective refrigeration capacities (RC) were 60.68, 59.28, and 57.72J·kg^−1^, with a slight decrease along the series. The experimental results show that the doping of La results in decreased T_C_, basically unchanged magnetic entropy, and slightly decreased RC.

## 1. Introduction

Heusler alloy is an intermetallic compound characterized by a highly ordered arrangement of atoms. It can be divided into full Heusler alloy and half-Heusler alloy, which provides the material with rich physical properties and application functions [1]. Heusler alloy has not only the properties of a metal alloy but also significant magnetic properties [2,3,4,5]. MM’X alloy is a kind of Heusler alloy, of which the MM’X alloy MnCoGe has attracted wide attention [6,7,8,9,10,11,12,13,14]. MnCoGe has an orthogonal TiNiSi-type (space group Pnma(64)) structure at room temperature. As the temperature increases, the alloy undergoes a magnetic phase transition at ~345 K [15]. Specifically, at 650 K, the first order structure phase transitions from an orthogonal TiNiSi phase to a high-temperature hexagonal Ni_2_In-type (space group P6_3_/mmc (194)) [15], and the Curie temperature is $T_{C}^{H}$ −275 K [16]. During the alloy cooling process, there is a phase transformation from hexagonal to orthorhombic that occurs, which is a non-diffusion type of lattice distortion referred to as a martensitic transformation. It can be seen that the magnetic phase transition and structural phase transition in MnCoGe alloy are separate processes. In order to increase the amount of change in magnetization during the phase transition process needed to obtain a large magnetocaloric effect, it is necessary to adjust the structural phase transition temperature to a point between the two-phase Curie temperature window to couple the structural phase transition with the magnetic transition.

In recent years, many studies have been conducted on the phase transition control of the magnetic structure of MnCoGe-based alloys. It has been shown that the martensitic phase transition temperature of MnCoGe-based alloys is more sensitive to pressure and chemical composition. The doping of trace elements or the application of pressure can be effective in significantly regulating the martensite transformation temperature. At present, there are four main methods for regulating the phase transition temperature of MnCoGe-based alloy Martensite: interstitial atom doping [13], transition element and/or main group element absence (excess) [5,17], transition element and/or main group element substitution [7], and external application pressure [8,18,19].

In 2017, Hassan et al. reduced the phase transition temperature of MnCoGe_1+x_ alloy by adding excessive main group element Ge and realized a magnetic and structure coupled phase transition [17]. Trung et al. first reported the giant magnetocaloric effect of MnCoGe alloy in 2010. The alloy is doped with B atoms and with the increase in B element doping, the martensite transformation temperature is reduced and the giant magnetocaloric effect is realized under a certain composition [20]. Liu et al. calculated the total energies of Mn vacancies and co-vacancies in the Ni_2_In structure by first principles (ab initio method), and the results confirmed that the co-vacancy structure (Mn_1−x_Co_x_)(Co_1−x*x_)Ge is energetically favorable. Therefore, by introducing Mn vacancies into the system, the coupling of magnetic and structural phase change was successfully realized by using the favorable Co vacancy, and a phase change window of about 100 K was found [21]. In 2016, Bao Lifu et al. realized magnetic and structure coupled phase transition and the resulting giant magnetocaloric effect in the MnCoGe_1−x_ alloy through the absence of the non-magnetic atom Ge [9]. In 2014, Shen et al. found that the substitution of Zn for Mn led to the coupling of phase transitions in the magnetic structure, and a large room temperature magnetocaloric effect was obtained. The magnetic entropy of the Mn_0.96_Zn_0.04_CoGe alloy reached a peak value of 4.3 J·kg^−1^K^−1^ when the magnetic field was changed from 0 to 0.1 T [6].

At the same time, rare earth permanent magnet materials also have excellent properties and are widely used in many fields. However, rare earth permanent magnets use a large amount of only Pr, Nd, Tb, and Dy, which leads to the unbalanced utilization of rare earth resources. Pr, Nd, Tb, and Dy resources are scarce, while La and Ce resources are abundant and overstocked. Therefore, in recent years, the development of magnetic materials containing La and Ce has become the focus of attention in academia and industrial production [22,23,24,25].

As an important half-Heusler alloy, MnCoGe has been studied in significant detail. The MnCoGe Heusler alloy can be applied to refrigeration equipment and zero-expansion materials due to its unique magnetic refrigeration, magnetocaloric and negative thermal expansion effects. It has huge development potential [26,27,28]. However, most of the samples currently studied are not single-phase, and most of the research samples are two-phase. There are few studies on the doping of high-temperature Ni_2_In-phase MnCoGe single-phase samples, and the doping of rare earth La atoms in high-temperature Ni_2_In-phase MnCoGe alloy has not previously been studied. As such, the study of the magnetic properties of La-doped high-temperature Ni_2_In phase MnCoGe alloy is meaningful for its potential application.

## 2. Experimental Method

In this experiment, MnCoGe_1−x_La_x_ (x = 0, 0.01, 0.03) were prepared using a high-temperature melting method. The purities of the raw materials Mn, Co, Ge, and La were 99.9%, 99.99%, 99.9999%, and 99.95%, respectively. Under the protection of pure argon (argon purity is 99.9999%) and using a vacuum electric arc furnace for smelting, the raw materials were smelted four times in order to ensure that the composition of the alloy ingots were uniform. Then, the ingots were placed in a quartz tube under vacuum at 1373 K for 72 h, followed by quenching with a mixture of ice and water.

A Rigaku D/max-2500 X-ray diffractometer with CuKα radiation source was used to analyze sample purity. The Rietveld method in FullProf program was used to refine the crystal structure of the sample. The magnetic properties of the samples were measured using a comprehensive physical property measurement system (PPMS; Quantum Design, USA), in which the temperature range and maximum magnetic field intensity were 5–400 K and ±5 T; and a vibrating sample magnetometer (model Lake Shore 7410), in which the temperature range and maximum magnetic field intensity were 100–420 K, and ±1.6 T.

## 3. Results and Discussion

**Figure 1:**
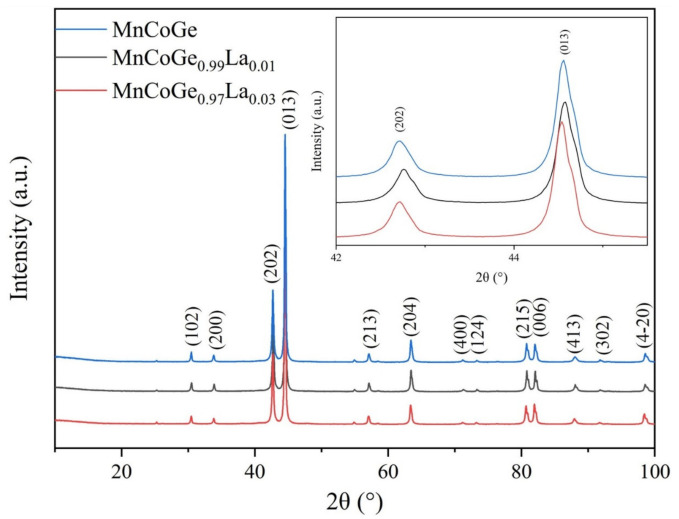
XRD pattern of MnCoGe_1−x_La_x_ alloy at room temperature.

Figure 1 shows the powder XRD patterns of the MnCoGe_1−x_La_x_ (x = 0, 0.01, 0.03) samples measured at room temperature. The XRD data of the MnCoGe_1−x_La_x_ series alloy samples were analyzed using Jade5.0 software. The results show that the samples were single phase after annealing. The structure of the single phase is a high-temperature Ni_2_In-type MnCoGe phase and the space group is P6_3_/mmc (194). These results are in good agreement with those reported by the Central Research Department [27]. The inset in Figure 1 is a partial enlarged view of sample XRD at 42–45.5°. As the figure shows, the positions of the strongest peak and the second strongest peak shift to a low angle with the increase in doped content, which reflects the increase in lattice that occurs with the increase in doped content.

**Figure 2:**
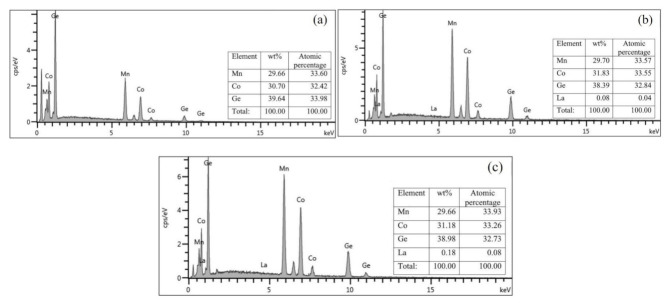
The EDS patterns of MnCoGe_1−x_La_x_ alloy: (**a**) MnCoGe; (**b**) MnCoGe_0.99_La_0.01_; (**c**) MnCoGe_0.97_La_0.03_.

In order to further determine the composition of the sample, MnCoGe_1−x_La_x_ (x = 0, 0.01, 0.03) series of alloys were analyzed by SEM/EDS. Figure 2 presents the EDS patterns of MnCoGe_1−x_La_x_ alloys. According to the analysis results on the EDS data obtained from scanning electron microscope, seen in Figure 2, this series of alloys are single phase, which agreed well with the results of XRD. It can be seen that the sample in Figure 2a with the composition Mn33.60Co32.42Ge33.98 was determined to be MnCoGe and that in Figure 2b with the composition Mn33.57Co33.55Ge32.84La0.04 was assigned to be MnCoGe_1−x_La_x_ (x = 0.01). The composition of the sample in Figure 2c is Mn33.96Co33.26Ge32.73La0.08, indicating the sample contained the single phase of MnCoGe_1−x_La_x_ (x = 0.03). These results are all in good agreement with those of XRD analysis.

The crystal structure parameters of the high-temperature Ni_2_In type MnCoGe (space group P6_3_/mmc (194)), in which the La atoms replace part of the Ge atom positions, were used as the initial data, and were input into the computer program FullProf. The peak shape function of the fitting correction is the pseudo-Voigt function. A total of 25 parameters were refined during Rietveld refinement, including lattice parameters, half-width, preferred orientation factor, symmetry, and isotropic temperature factor. Finally, the Rietveld refinement results of the MnCoGe_1−x_La_x_ series alloy samples were obtained.

**Figure 3:**
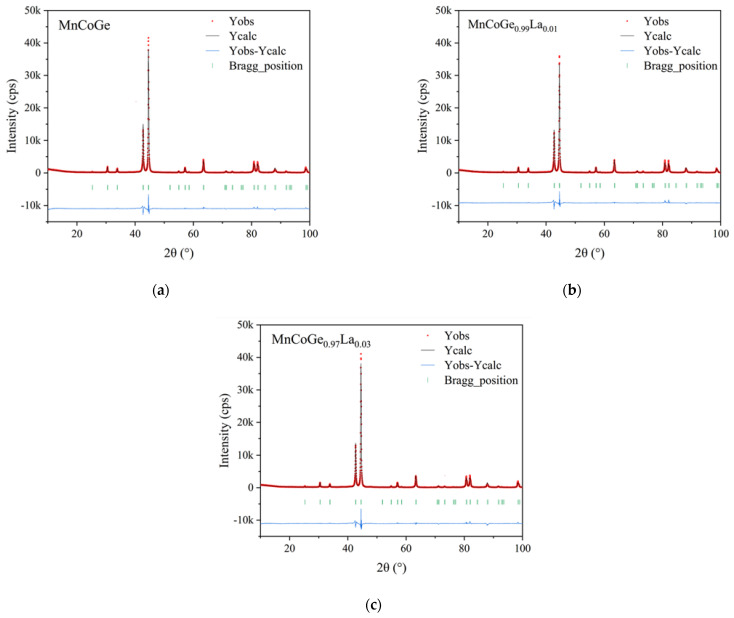
Rietveld refinement results for MnCoGe_1−x_La_x._: (**a**) MnCoGe; (**b**) MnCoGe_0.99_La_0.01_; (**c**) MnCoGe_0.97_La_0.03_.

Figure 3 shows the Rietveld refinement pattern of the MnCoGe_1−x_La_x_ series alloy samples. From this pattern, the experimental values, calculated values, and residuals can be observed. The reliability of the Rietveld refinement results is reflected by the residual variance factor R_P_ of the graph and the residual variance factor R_WP_ of the weighted graph. The expression is:

(1) $$R_{P}=\frac{\sum Y_{i}\left(\mathrm{obs}\right)-Y_{i}\left(\mathrm{calc}\right)}{\sum Y_{i}\left(\mathrm{obs}\right)}$$

(2) $$R_{WP}={\left\{\frac{\sum_{{}_{wi}}[Y_{i}{\left(\mathrm{obs}\right)}^{2}-Y_{i}{\left(\mathrm{calc}\right)}^{2}}{\sum_{{}_{wi}}{\left[Y_{i}\left(\mathrm{obs}\right)\right]}^{2}}\right\}}^{1/2}$$

The Rietveld refinement effect can be judged by the size of the residual. The smaller the R_P_ and R_WP_ values, the more reliable the results. The Rietveld refinement results of the MnCoGe_1−x_La_x_(x = 0, 0.01, 0.03) samples are shown in Table 1. From the values of R_P_, R_WP_, and R_EXP_ in Table 1, it can be observed that the Rietveld refinement results are credible. As the doped amount increases, the lattice constants a, b, and c of the high-temperature Ni_2_In-type MnCoGe gradually increase. The unit cell volume also gradually increases. This is because the La atomic radius (1.87 Å) is larger than the Ge atomic radius (1.40 Å), which causes the crystal lattice to become larger. Table 2 shows the results of the site occupation and the occupancy of various atoms in refined MnCoGe_1−x_La_x_.

**Figure 4:**
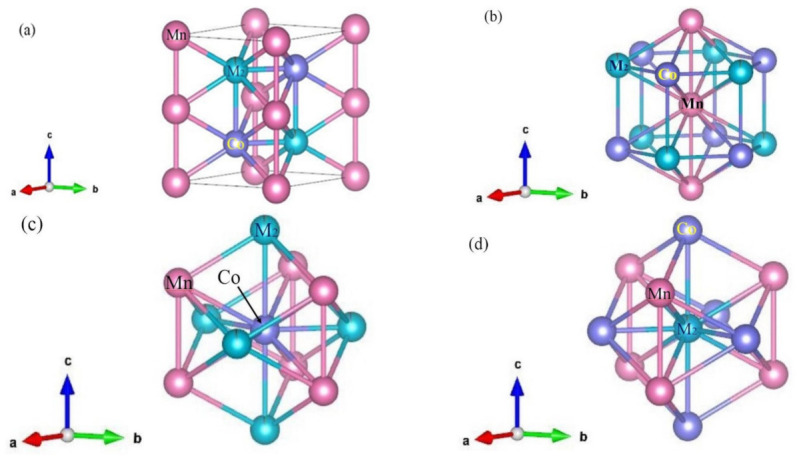
MnCoGe_0.97_La_0.03_ crystal structure: (**a**) the crystal structure of the MnCoGe_0.97_La_0.03_; (**b**) the atomic environments of Mn; (**c**) Co and (**d**) M_2_.

Figure 4a shows the crystal structure of the MnCoGe_0.97_La_0.03_ alloy. The pink and purple spheres in the figure are Mn and Co atoms, and the dark green and green spheres are M_2_ atoms. The green part is Ge atoms, and the dark green part is doped La atoms. Figure 4b–d are the atomic environments of Mn, Co, and M_2_ atoms. As can be observed, the Mn atom is linked with two adjacent Mn atoms, six adjacent Co atoms, and six adjacent M_2_ atoms. Six adjacent Mn atoms and five adjacent M_2_ atoms are connected with the central Co atom. The Mn atom is connected to six adjacent Mn atoms and five adjacent Co atoms. From Table 3, it can also be observed that the bond length changes after refinement. It can also be argued that the Mn–Mn and Mn–Co distances become longer due to increasing doped La content.

In this study, the magnetic properties of MnCoGe_1−x_La_x_ (x = 0, 0.01, 0.03) series alloy samples were measured using a comprehensive physical property measurement system (PPMS; Quantum Design, USA) and vibrating sample magnetometer (model Lake Shore 7410).

**Figure 5:**
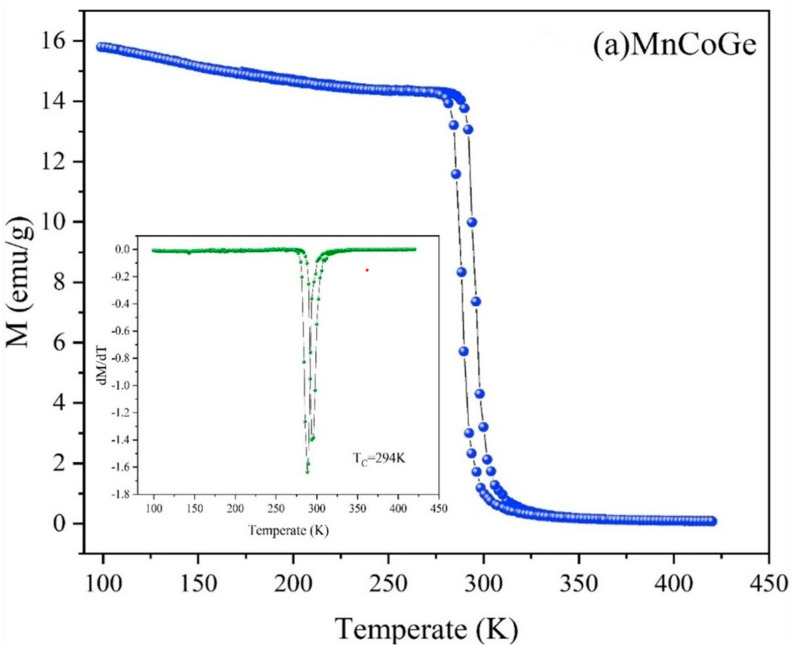

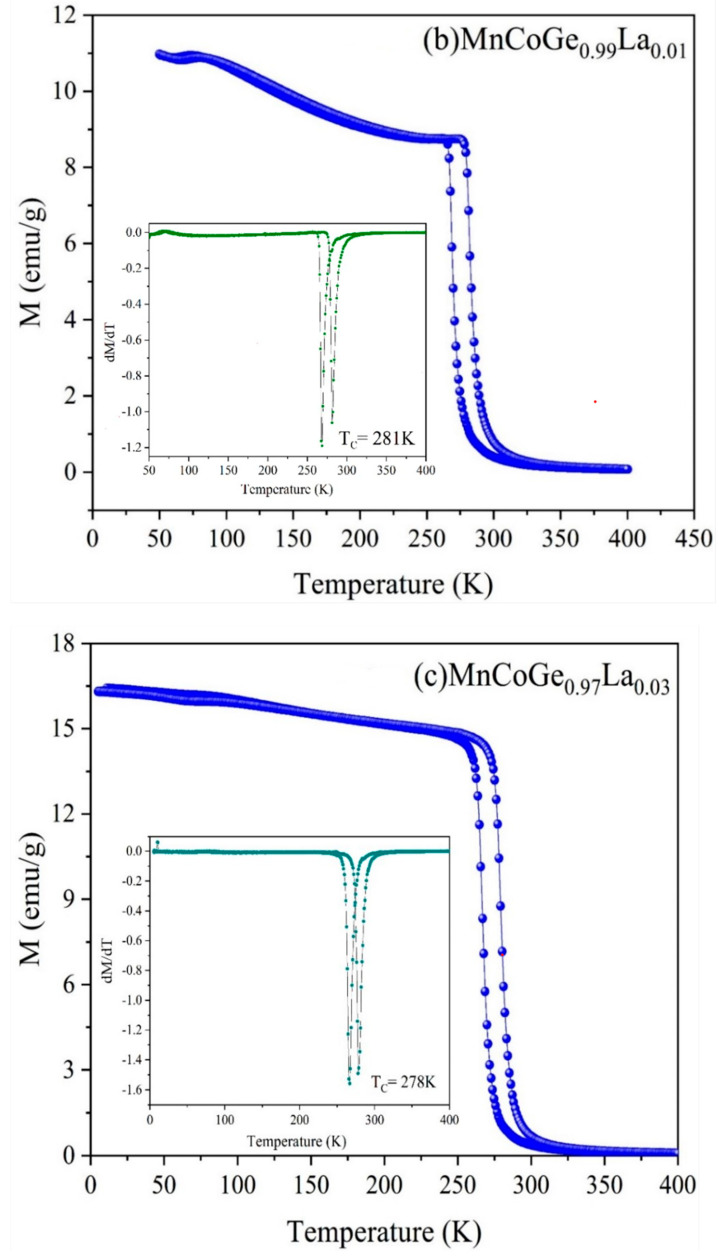
Temperature dependence of magnetization of (**a**) MnCoGe, (**b**) MnCoGe_0.99_La_0.01_, and (**c**) MnCoGe_0.97_La_0.03_ measured under a magnetic field of 500 Oe.

Figure 5 shows the temperature dependence of the magnetization (M) of MnCoGe_1−x_La_x_ series alloy samples in an external magnetic field of 0.05 T (MT curves). Here, T_C_ is defined as the temperature at which the maximum slope occurs during zone field cooling (FC), where Tc represents the general meaning of the T_C_ that is the transition temperature between the FM and PM phases. The inset in the figure shows the spectrum of the dM/dT curve. The peak position on the right is Tc. When x = 0, 0.01, and 0.03, the corresponding Tc values of MnCoGe_1−x_La_x_ alloys are 294, 281, and 278 K, respectively. With an increase in doped La content, the Curie temperature shows an obvious downward trend. An obvious thermal hysteresis in the field cooling (FC) and field cooling heating (FCH) curves near the Curie temperature can be clearly observed. This thermal hysteresis is caused by the martensitic transformation in the MnCoGe_1−x_La_x_ series alloys, which confirms the first-order nature of the transformation. Thermal hysteresis also shows that the magnetic structure transition is not coupled.

**Figure 6:**
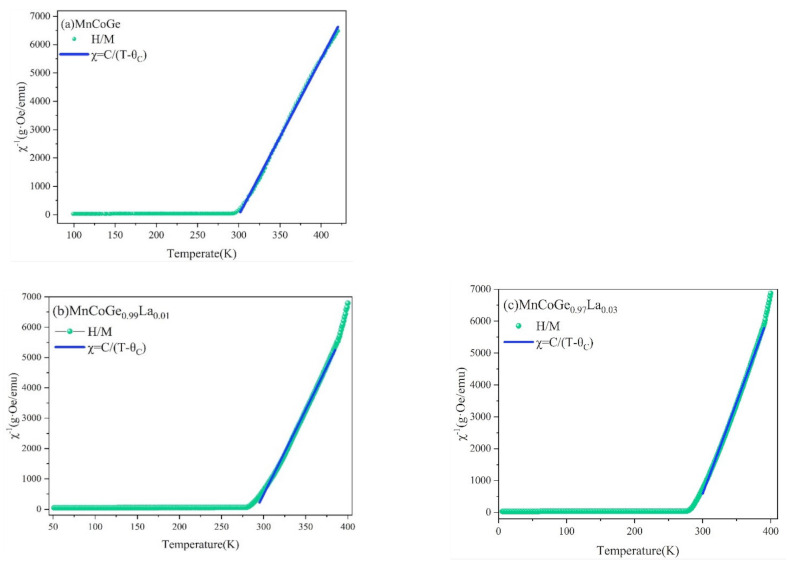
χ^−1^–T fitting curves of MnCoGe_1−x_La_x_: (**a**) MnCoGe; (**b**) MnCoGe_0.99_La_0.01_; (**c**) MnCoGe_0.97_La_0.03_.

The curve of χ^−1^~T is shown in Figure 6. It can be found that the reciprocal of the magnetic susceptibility of MnCoGe_1−x_La_x_ (x = 0, 0.01, 0.03) alloys in the high-temperature region varies linearly with temperature, indicating that these compounds obey the Curie–Weiss law:

(3) $$\chi =\frac{C}{T-\theta_{C}}.$$

The green lines in Figure 6 are the curve of the reciprocal of the magnetic susceptibility with temperature, and the blue lines are the Curie–Weiss fitting. The Curie constant *C* and the Curie–Weiss temperature *θ_C_* can be calculated separately by fitting the curve linearly.

From the fitting curve analysis of Figure 6, the Curie constants of MnCoGe_1−x_La_x_ can be obtained as 0.0181 (2), 0.0179 (2), and 0.0174(3), respectively. Moreover, the Curie–Weiss temperatures *θ_C_* of MnCoGe_1−x_La_x_ are 300.36, 291.36, and 289.97 K, respectively. The expression of the Curie constant *C* is as follows:

(4) $$C=\frac{N_{A}\mu_{B}^{2}}{3{Κ}_{B}}\mu_{\mathrm{eff}}^{2}.$$

Among the various constants in the expression are the Avogadro constant, *N_A_* = 6.023 × 10^23^ mol^−1^; the Bohr constant, *μ_B_* = 9.274 × 10^−21^ emu; and the Boltzmann constant, *k_B_* = 1.380649 × 10^−23^(J·K^−1^). The reciprocal of the magnetic susceptibility χ^−1^ and the reciprocal of the slope of the linear part of the temperature (T) curve in the high-temperature region are the Curie constant C. The above formula can be simplified to:

(5) $$\mu_{eff}\left(\mu_{B}\right)=\sqrt{8.0028\times C\times M}$$

where *M* is the relative molecular mass. By substituting the fitted values into the formula, it can be calculated that the effective magnetic moments of MnCoGe_1−x_La_x_ are 5.20_,_ 5.17, and 5.12 μ_B_, respectively. It can also be observed that, with the doping of La, the effective magnetic moment gradually decreases. Due to La doping, the Mn–Mn distance in the compound becomes larger, which weakens the interaction between Mn atoms. Wang et al. [16,29] demonstrated that the smaller Mn–Mn distance separation in hexagonal Ni_2_In-type MnCoGe-based alloys results in a wider 3d band width and less exchange split between the majority band and minority bands. Therefore, the moment of the system is generally smaller. Moreover, it has been proven that by changing the intercrystalline bond length of the material, the generated three-dimensional band can reduce electron splitting exchange interaction, thereby reducing the net magnetic moment.

Table 4 shows the values of various parameters obtained after the Curie–Weiss fitting of MnCoGe_1−x_La_x_. From the comparison between the Curie–Weiss temperature and the Curie temperature in the table, it is clear that the two temperature values are close. It can also be seen that the fitting results are reasonable.

**Figure 7:**
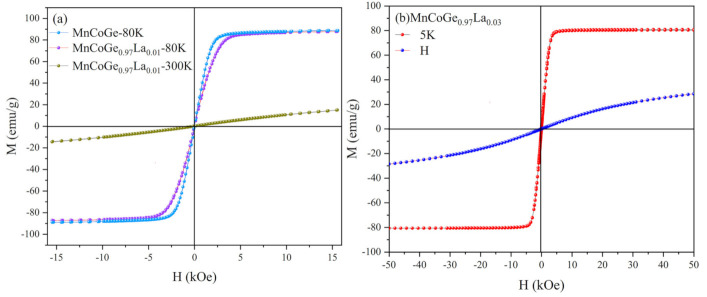
M-H curves of MnCoGe_1−x_La_x_ measured at different temperatures: (**a**) MnCoGe and MnCoGe_0.99_La_0.01_; (**b**) MnCoGe_0.97_La_0.03_.

It can be seen from Figure 7a that the MnCoGe_1−x_La_x_ (x = 0.01) alloy is in a paramagnetic state at 300 K and in a ferromagnetic state at 80 K. From Figure 7b, the sample MnCoGe_1−x_La_x_ (x = 0.03) is not completely converted to paramagnetism at 300 K, but still has ferromagnetic behavior. According to Bohol’s law [30], one can draw the M–H^−1^ curve and extrapolate the linear part to H^−1^ = 0 to obtain the saturation magnetization of M_S_. As can be seen from Figure 7a, the saturation magnetizations of MnCoGe and MnCoGe_0.99_La_0.01_ at 80 K are 88.75 and 87.55 emu/g, respectively, and there is no coercivity. In Figure 7b, it is apparent that the saturation magnetization of MnCoGe_0.97_La_0.03_ at 5 K is 80.91 emu/g. No coercivity was observed in this series of compounds, indicating that the compounds can be used as soft magnetic materials.

**Figure 8:**
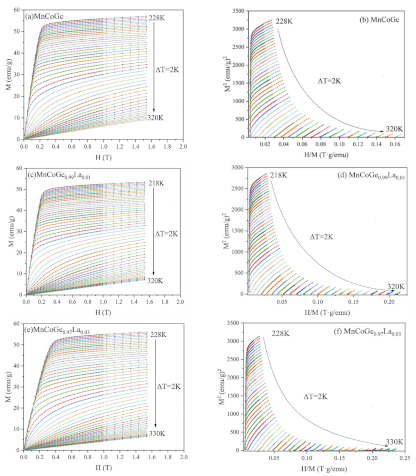
Isothermal magnetization curves (**a,c,e**) and Arrottt curves (**b,d,f**) of MnCoGe_1−x_La_x_ at different temperatures.

The isothermal magnetization curve (MH) of MnCoGe_1−x_La_x_ series alloys was measured, and the temperature ranges are 228–320, 218–320, and 228–330 K. The temperature interval is 2 K, as shown in Figure 8a,c,e. Figure 8b,d,f shows the corresponding Arrott curve (M^2^–H/M) and the Arrott curve made from the isothermal magnetization (M–H) curve, which can be used as the basis for evaluating the magnetic phase change. Experimentally, the type of magnetic phase transition can be determined by the slope in the Arrott curve. Generally speaking, a positive slope corresponds to a second-order phase transition, and a negative slope corresponds to a first-order phase transition. In this work, all Arrott curves show a positive slope near the Curie temperature, revealing the characteristics of the second-order phase transition (SOMT) based on the Banerjee criterion [30]. Magnetic refrigeration materials require a secondary phase change from FM (ferromagnetic) to PM (paramagnetic) because the primary phase change is accompanied by hysteresis loss. It is apparent in the results that MnCoGe_1−x_La_x_ series alloys meet this characteristic.

According to the related theories of thermodynamics, the magnetocaloric effect of materials can be calculated by using the Maxwell relationship:

(6) $${\left(\frac{\partial M}{\partial T}\right)}_{H}={\left(\frac{\partial S}{\partial H}\right)}_{T}$$

Isothermal magnetic entropy change is obtained by the isothermal magnetization curve, which can be described by the following formula:

(7) $$\Delta S_{M}(T,H_{\max})=S_{M}(T,H_{\max})-S_{M}(T,0)=\int_{0}^{H_{\max}}{\left(\frac{\partial M}{\partial T}\right)}_{H}dH$$

Here, *S*, *M*, *H,* and *T* represent entropy, magnetization, magnetic field, and temperature, respectively. A series of isothermal magnetic entropy changes around the phase transition temperature of the sample can be approximated as:

(8) $$\Delta S_{M}\left(T,H\right)=\sum_{i}\frac{M_{i+1}\left(T_{i+1},H\right)-M_{i}\left(T_{i},H\right)}{T_{i+1}-T_{i}}\Delta H$$

**Figure 9:**
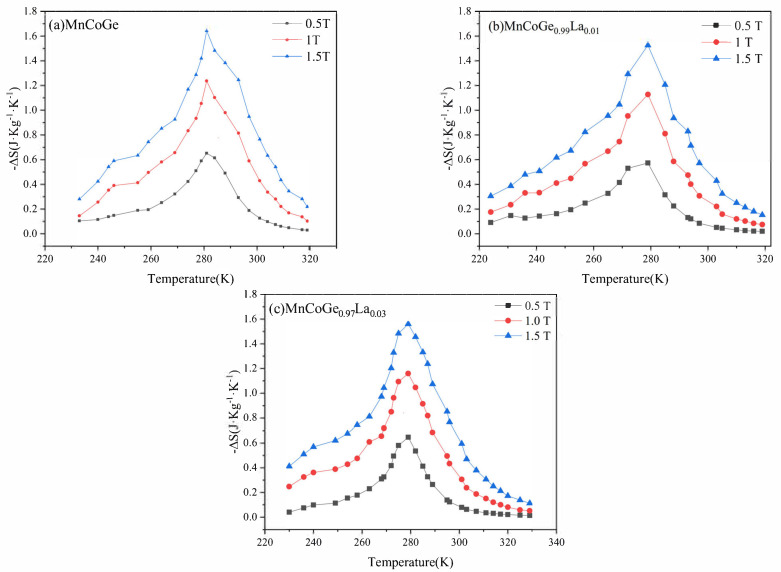
Temperature dependence of the magnetic entropy changes at different amplitudes of change in the applied magnetic field for MnCoGe_1−x_La_x_: (**a**) MnCoGe, (**b**) MnCoGe_0.99_La_0.01_, (**c**) MnCoGe_0.97_La_0.03_.

Using Maxwell’s relationship, the isothermal magnetic entropy change in the second-order phase change material is calculated from the isothermal magnetization curve. Figure 9 shows the results of isothermal magnetic entropy changes in MnCoGe_1−x_La_x_ alloy magnetization. The magnetic entropy changes in MnCoGe, MnCoGe_0.99_La_0.01_, and MnCoGe_0.97_La_0.03_ change the most near the magnetic transition temperature, while they increase with the increase in the external magnetic field. Under an external magnetic field of 1.5T, the maximum magnetic entropy changes $\left|\Delta S_{M}\right|$ of MnCoGe, MnCoGe_0.99_La_0.01_, and MnCoGe_0.97_La_0.03_ are 1.64, 1.53, and 1.56 J·kg^−1^·K^−1^.

It can be seen that the magnetic entropy changes in the alloy samples have no obvious changes in relation to the doping of La atoms, as they were decreased compared to MnCoGe_0.95_Ga_0.05_ [31] and MnCoGe_0.98_Al_0.02_ [32], among others. However, the specific mechanism is not clear. The reduction in transition temperature and magnetic entropy change may be the result of a combination of many factors. The change in the covalent bond and local environment could be the cause of the decrease in $\left|\Delta S_{M}\right|$ [33]. In addition, doping the Ge site with a less electronegative La element will result in a stronger Mn–Mn bond (Pauling—electronegativity of Ge and La are 2.01 and 1.158, respectively) [34], thereby reducing the transition temperature and magnetic entropy change, which is similar in the cases of MnCo_1−x_Cd_x_Ge [33], MnCoGe_1−x_Si_x_ [34], and MnCoGe_1−x_Al_x_ [32].

Although the basic requirement for magnetic refrigeration materials is a large magnetic entropy change, a material with a large magnetic entropy change $\left|\Delta S_{M}\right|\;$cannot guarantee high refrigeration efficiency. The recognized standard for evaluating refrigeration efficiency is refrigeration capacity (RC), which represents the amount of heat transfer in a thermodynamic cycle. The value of refrigeration capacity (RC) is measured by different methods. In this article, RC value is calculated using the method proposed by Gschneidner et al. based on the following formula:

(9) $$\mathrm{RC}=\overset{T_{hot}}{\underset{T_{cold}}{\int }}\Delta S\left(T\right)dT$$

In the formula, *T_hot_* and *T_cold_* correspond to the temperature range of $\left|\Delta S_{M}\right|$, which is half of the highest peak value. The value of the temperature span of the entire width, corresponding to a half-maximum (FWHM) value of $\left|\Delta S_{M}\right|$, is the difference between *T_hot_* and *T_cold_* (expressed as δT_FWHM_, where δT_FWHM_ = *T_hot_* − *T_cold_*). Under a magnetic field of 1.5 T, the δT_FWHM_ values of the $\left|\Delta S_{M}\right|$ for the MnCoGe_1−x_La_x_ alloys are 37, 39, and 37 K for x = 0, 0.01, and 0.03, respectively. For ΔH = 1.5 T, the refrigeration capacity (RC_FWHM_) values for the MnCoGe_1−x_La_x_ alloys are 60.68, 59.28, and 57.72J·kg^−1^ for x = 0, 0.01, and 0.03, respectively. For ΔH = 1 T, the refrigeration capacity (RC_FWHM_) values for the MnCoGe_1−x_La_x_ alloys are 38.44, 36.16, and 34.8J·kg^−1^ for x = 0, 0.01, and 0.03, respectively. Table 5 shows MCE of MnCoGe_1−x_La_x_ alloys compared with other materials.

## 4. Conclusions

In this work, MnCoGe_1−x_La_x_ (x = 0, 0.01, 0.03) series alloys are all in the high-temperature Ni_2_In-type MnCoGe phase. In MnCoGe_1−x_La_x_, the lattice constant gradually increases and the unit cell volume becomes larger with an increase in doped La content.

The magnetic properties of MnCoGe_1−x_La_x_ series alloys were systematically studied, and we found that MnCoGe_1−x_La_x_ is ferromagnetic at low temperatures and paramagnetic at room temperature. With an increase in doped content, the T_C_ decreased and the T_C_ of the MnCoGe_1−x_La_x_ series alloys were 294, 281, and 278 K, respectively. With an increase in doped La content, the saturation magnetization at 80 K decreased slightly; being 88.75 emu/g for MnCoG, 87.55 emu/g for MnCoGe_0.99_La_0.01_, and 80.91 emu/g for MnCoGe_0.97_La_0.03_ at 5K. The maximum magnetic entropy changes in MnCoGe_1−x_La_x_ series alloys at $\left|\Delta S_{M}\right|$1.5T were 1.64, 1.52, and 1.56 J·kg^−1^·K^−1^, respectively, and the refrigeration capacities (RC) were 60.68, 59.28, and 57.72 J·kg^−1^, respectively, with a slight decrease along the series. These changes can be observed in our study, the findings of which can serve as a reference for future research into the application of rare earth element-doped MnCoGe series alloys in magnetic refrigeration.

## Figures and Tables

**Table 1:** Rietveld refinement result for MnCoGe_1−x_La_x_ series alloy.

Compounds	MnCoGe	MnCoGe_0.99_La_0.01_	MnCoGe_0.97_La_0.03_
Space group	P6_3_/mmc (194)		
Radiation	CuKα		
Unit cell parameters			
a/(Å)	4.0641(1)	4.0648(1)	4.0695(1)
b/(Å)	4.0641(1)	4.0648(1)	4.0695(1)
c/(Å)	5.2882(2)	5.2905(2)	5.2980(2)
Lattice volume V(Å3)	87.337(5)	87.430(7)	87.761(2)
Reliability factors			
R_p_ (%)	12.9	13.3	13.0
R_WP_ (%)	15.5	15.7	15.7
R_exp_ (%)	5.14	4.96	4.96

**Table 2:** Atomic site occupation and occupancy rate of MnCoGe_1−x_La_x_.

Doping Content x	Atoms	Site Occupation	x	y	z	Occupancy
0	Mn	2a	0	0	0	1
	Co	2d	1/3	2/3	3/4	1
	Ge	2c	1/3	2/3	1/4	1
0.01	Mn	2a	0	0	0	1
	Co	2d	1/3	2/3	3/4	1
	M_1_ *	2c	1/3	2/3	1/4	0.993Ge + 0.007La
0.03	Mn	2a	0	0	0	1
	Co	2d	1/3	2/3	3/4	1
	M_2_ **	2c	1/3	2/3	1/4	0.976Ge + 0.024La

* M_1_ = 0.993Ge + 0.007La; ** M_2_ = 0.976Ge + 0.024La.

**Table 3:** The number of different atoms of each central atom and its nearest neighbor.

Central Atom	Connected to the Atom	Atomic Number	Distance (Å)		
			0	0.01	0.03
Mn	Mn	2	2.6441(10)	2.6453(10)	2.6490(10)
	Co	6	2.6930(7)	2.6937(8)	2.6970(8)
	Ge/M_1_/M_2_	6	2.6930(7)	2.6937(8)	2.6970(8)
Co	Mn	6	2.6930(7)	2.6937(8)	2.6970(8)
	Ge/M_1_/M_2_	5	2.3462(6)	2.3466(8)	2.3493(8)
Ge/M_1_/M_2_	Mn	6	2.6930(7)	2.6937(8)	2.6970(8)
	Co	5	2.3462(6)	2.3466(8)	2.3493(8)

**Table 4:** Magnetic ordering temperature, T_C_; Curie–Weiss temperatures, θ_C_; Curie–Weiss constant, C; and effective moments, μ_eff_ for all MnCoGe_1−x_La_x_ compounds.

Doping Content x	T_C_/K	θ_C_/K	C	Effective Magnetic Moment/(μ_B_/Formula)
0	294	300.36(2)	0.0181(2)	5.20
0.01	281	291.36(5)	0.0179(2)	5.17
0.03	278	289.97(3)	0.0174(3)	5.12

**Table 5:** The MCE of MnCoGe_1−x_La_x_ alloys compared with other materials.

Materials	Magnetic Field/T	$\left|\Delta S_{M}\right|/J\cdot {\mathrm{kg}}^{-1}\cdot K^{-1}$	T_pk_/K	RC/J·kg^−1^	δT_FWHM_	Reference
MnCoGe	1	1.24	281	38.44	31	This work
	1.5	1.64	281	60.68	37	
MnCoGe_0.99_La_0.01_	1	1.13	279	36.16	32	This work
	1.5	1.52	279	59.28	39	
MnCoGe_0.97_La_0.03_	1	1.16	279	34.80	30	This work
	1.5	1.56	279	57.72	37	
MnCoGe_0.5_Si_0.5_	5	4.4	373	282	96	[34]
Mn_0.97_Fe_0.03_CoGe	5	9	276	221	31	[35]
Mn_0.96_Fe_0.04_CoGe	5	5	247	261	28	[35]
Gd_5_Si_2_Ge_2_	5	5.8	300	305	/	[36]

## Data Availability

Not applicable.
